# Nuclear Respiratory Factor 1 Mediates the Transcription Initiation of *Insulin-Degrading Enzyme* in a TATA Box-Binding Protein-Independent Manner

**DOI:** 10.1371/journal.pone.0042035

**Published:** 2012-08-03

**Authors:** Lang Zhang, Qingyang Ding, Zhao Wang

**Affiliations:** Protein Science Key Laboratory of the Ministry of Education, Department of Biological Sciences and Biotechnology, School of Medicine, Tsinghua University, Beijing, China; Omaha Veterans Affairs Medical Center, United States of America

## Abstract

CpG island promoters often lack canonical core promoter elements such as the TATA box, and have dispersed transcription initiation sites. Despite the prevalence of CpG islands associated with mammalian genes, the mechanism of transcription initiation from CpG island promoters remains to be clarified. Here we investigate the mechanism of transcription initiation of the CpG island-associated gene, *insulin-degrading enzyme* (*IDE*). *IDE* is ubiquitously expressed, and has dispersed transcription initiation sites. The *IDE* core promoter locates within a 32-bp region, which contains three CGGCG repeats and a nuclear respiratory factor 1 (NRF-1) binding motif. Sequential mutation analysis indicates that the NRF-1 binding motif is critical for *IDE* transcription initiation. The NRF-1 binding motif is functional, because NRF-1 binds to this motif *in vivo* and this motif is required for the regulation of *IDE* promoter activity by NRF-1. Furthermore, the NRF-1 binding site in the *IDE* promoter is conserved among different species, and dominant negative NRF-1 represses endogenous IDE expression. Finally, TATA-box binding protein (TBP) is not associated with the *IDE* promoter, and inactivation of TBP does not abolish *IDE* transcription, suggesting that TBP is not essential for *IDE* transcription initiation. Our studies indicate that NRF-1 mediates *IDE* transcription initiation in a TBP-independent manner, and provide insights into the potential mechanism of transcription initiation for other CpG island-associated genes.

## Introduction

DNA methylation, an epigenetic modification that regulates chromatin structure and gene expression [1], [2], occurs predominantly at cytosines of CpG dinucleotides in vertebrates [3], [4]. There are three striking features of CpG sites in mammalian genomes. First, the majority (60%–90%) of CpG sites are methylated [5]. Second, mammalian genomes are depleted of CpG sites, a phenomenon called CG suppression. The observed rate of CpG sites is approximately one-fifth of the rate expected based on the GC content [6]. This rarity of CpG sites arises from the spontaneous mutation of methylated cytosines to thymidines by deamination, which converts CpG to TpG dinucleotides [7], [8]. Thirdly, unmethylated CpG sites cluster to form CpG islands [9]. CpG islands, which have a high GC content and a high observed-to-expected CpG ratio relative to the bulk genome, are often associated with mammalian gene promoters, including all housekeeping genes and some tissue-specific genes [10]. Actually, CpG islands serve as an important criterion for gene prediction [11], [12], [13]. In human, it was estimated that approximately 72% of genes are associated with CpG islands [14].

The core promoter is the minimal promoter region necessary to direct transcription initiation. Several core promoter elements, including the TATA box, the Inr (initiator) element, the downstream core promoter element (DPE), the motif ten element (MTE) and TFIIB recognition elements (BREs), have been identified for RNA polymerase II-transcribed genes, and they function individually or cooperatively in recruiting the transcription apparatus to the promoter region [15]. Promoters with these core promoter elements initiate transcription at a focused start site, which varies by only a few nucleotides. However, CpG island promoters often lack these canonical core promoter elements and have dispersed transcription initiation sites over 50–150 nucleotides [16]. Although genes with focused promoters are well studied, the mechanism for transcription apparatus recruitment to CpG island promoters remains to be clarified.

Insulin-degrading enzyme (IDE) is a ubiquitously expressed zinc metalloprotease that degrades several substrates [17], including insulin and β-amyloid (Aβ) [18], [19]. IDE is closely related to diabetes mellitus (DM) and Alzheimer’s disease (AD), because insulin and Aβ play critical roles in the pathogenesis of DM and AD, respectively. GK rats, which are a well-characterized model for type II DM, have *IDE* missense mutations, and exhibit impaired insulin and Aβ degradation [20]. *IDE* knockout mice also show accumulation of endogenous Aβ, hyperinsulinemia and glucose intolerance [21]. However, the exact role of IDE in DM pathogenesis seems a little elusive. A recent study indicates that the diabetic phenotype of *IDE* knockout mice is an emergent compensatory response to chronic hyperinsulinemia, but not a direct consequence of IDE deficiency [22]. In addition, human genetic studies suggest that *IDE* polymorphisms are associated with the pathogenesis of both type II DM [23] and AD [24]. These results imply that IDE dysfunction may cause DM or AD and underline the importance of characterizing the transcriptional regulation of *IDE*.

In this article, we focus on the mechanism of *IDE* transcription initiation. The mouse *IDE* promoter contains a CpG island and has dispersed transcription initiation sites. Promoter deletion analysis indicates that the *IDE* core promoter contains a nuclear respiratory factor 1 (NRF-1) binding motif which is essential for transcription initiation. We further demonstrated that NRF-1 binds to the *IDE* promoter and dominant negative NRF-1 represses *IDE* transcription. Finally, we showed that TBP is not essential for *IDE* transcription initiation.

## Results

### Mapping the Core Promoter Region of Mouse *IDE*

**Figure 1:**
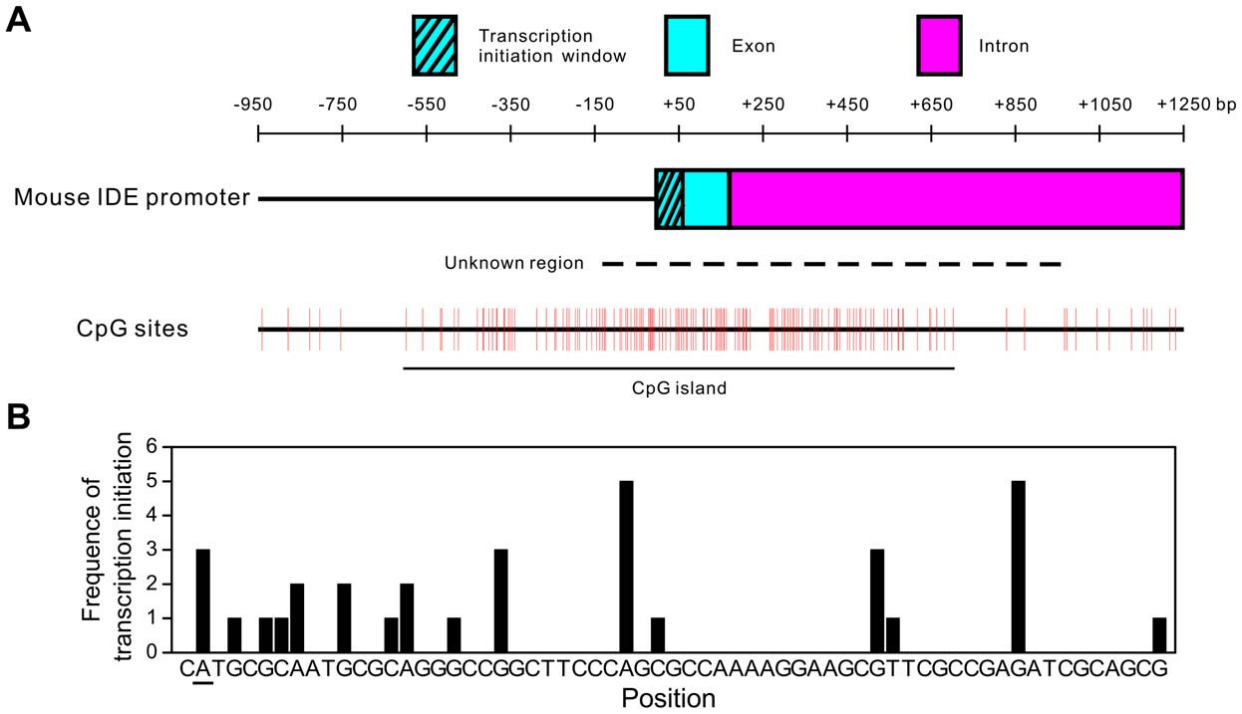
The mouse *IDE* promoter contains a CpG island and has dispersed transcription initiation sites. (**A**) Representation of the mouse *IDE* promoter. The dashed line indicates the unknown region which was cloned and sequenced in this study. The mouse *IDE* promoter contains a CpG island with a length of approximately 1300 bp. (**B**) The transcription initiation sites of mouse *IDE*. The frequency of transcription initiation from different sites is shown. The mouse *IDE* promoter has dispersed transcription initiation sites located within a window of 62 bp. The first transcription initiation site is underlined.

Because the sequence of the mouse *IDE* promoter was incomplete in the NCBI genomic database (Figure 1A), we cloned and sequenced the unknown region [Genbank: JN038396]. The mouse *IDE* promoter has a high CpG frequency and contains a CpG island approximately 1300 bp long (Figure 1A). The transcription initiation sites of mouse *IDE* were determined using the 5′-rapid amplification of cDNA ends (5′-RACE) method. The *IDE* promoter possesses dispersed transcription initiation sites located within a 62-bp region (Figure 1 A and B). *IDE* transcription starts with a purine at position +1 at a frequency of 91% and with a pyrimidine-purine dinucleotide at position −1,+1 at a frequency of 67%, which is consistent with previous studies showing that RNA polymerase II-mediated transcription is preferentially initiated at a pyrimidine-purine dinucleotide at position −1,+1 in mammals [16].

**Figure 2:**
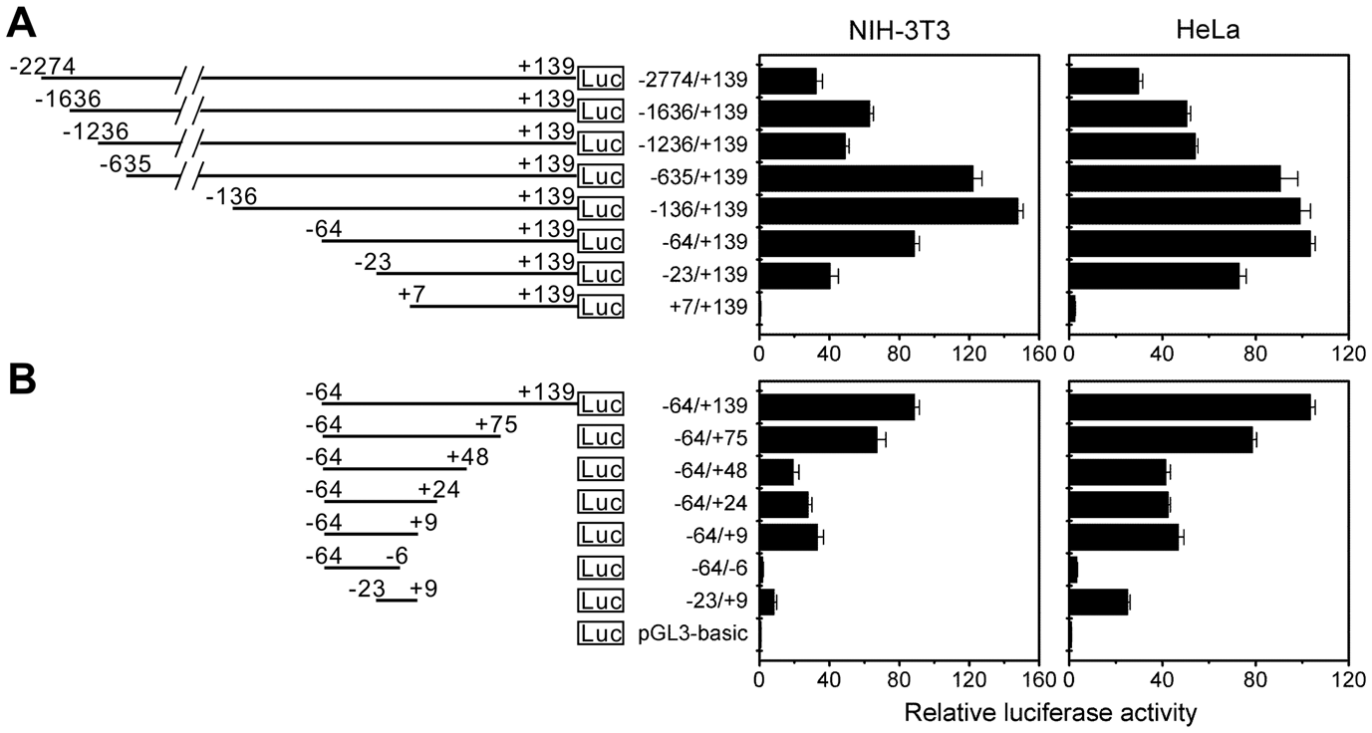
Mapping the core promoter region of mouse *IDE*. (**A**) and (**B**) The region between −23 and +9 of the mouse *IDE* promoter is essential for transcription initiation and behaves as the core promoter. Different truncations of the mouse *IDE* promoter were cloned into the pGL3-basic vector. NIH-3T3 or HeLa cells were transiently transfected with luciferase reporter plasmids of the mouse *IDE* promoter (0.8 µg) and *Renilla* luciferase reporter plasmid (pCMV-RL, 8 ng). Twenty-four hours after transfection, cells were lysed, and the luciferase activity was determined. Firefly luminescence signal was normalized based on the *Renilla* luminescence signal. Data are represented as fold of the firefly luciferase activity of the pGL3-basic vector.

Searching the mouse *IDE* promoter revealed no TATA box, Inr or other canonical core promoter elements around the transcription initiation sites. To determine the region that is essential for *IDE* transcription initiation, we constructed luciferase reporter plasmids of the mouse *IDE* promoter with different truncations at the 5′ or 3′ terminus. For convenience, we set the first transcription initiation site that we identified (Figure 1B) as position +1. Deleting the 5′ terminus of the mouse *IDE* promoter from −2774 to −23 retained its transcriptional activity, while further deletion to +7 abolished its transcriptional activity (Figure 2A). On the other hand, deleting the 3′ terminus of the mouse *IDE* promoter from +139 to +9 retained its transcriptional activity, while further deletion to −6 abolished its transcriptional activity (Figure 2B). These results indicate that the region between −23 and +9 of the mouse *IDE* promoter is essential for transcription initiation and behaves as the core promoter. Indeed, this region was capable of driving transcription initiation in both NIH-3T3 and HeLa cells (Figure 2B).

### The *IDE* Core Promoter Contains a Functional NRF-1 Binding Site that is Essential for Transcription Initiation

**Figure 3:**
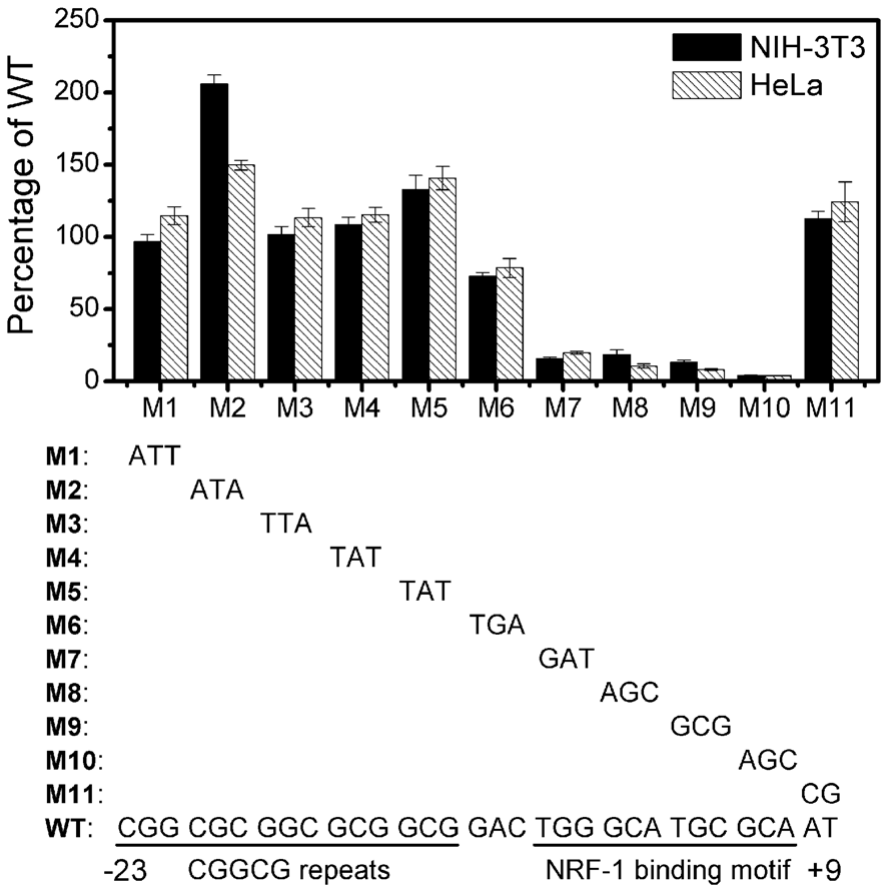
Sequential mutation of the mouse *IDE* core promoter. The mouse *IDE* core promoter contains three CGGCG repeats and a NRF-1 binding motif. The core promoter region with sequential mutations (M1 to M11) was cloned into the pGL3-basic vector. Luciferase activity of the reporter plasmids in NIH-3T3 and HeLa cells is represented as a percentage of the wild-type (WT) reporter plasmid.

Analysis of the region between −23 and +9 of the mouse *IDE* promoter revealed three CGGCG repeats and a NRF-1 binding motif (Figure 3). To identify which element is essential for *IDE* transcription initiation, we sequentially mutated this region and detected changes in its transcriptional activity. Mutations within the NRF-1 binding motif abolished transcription initiation, while mutations in other regions resulted in little or no decrease in the transcriptional activity (Figure 3), suggesting that the NRF-1 binding motif is critical for *IDE* transcription initiation.

**Figure 4:**
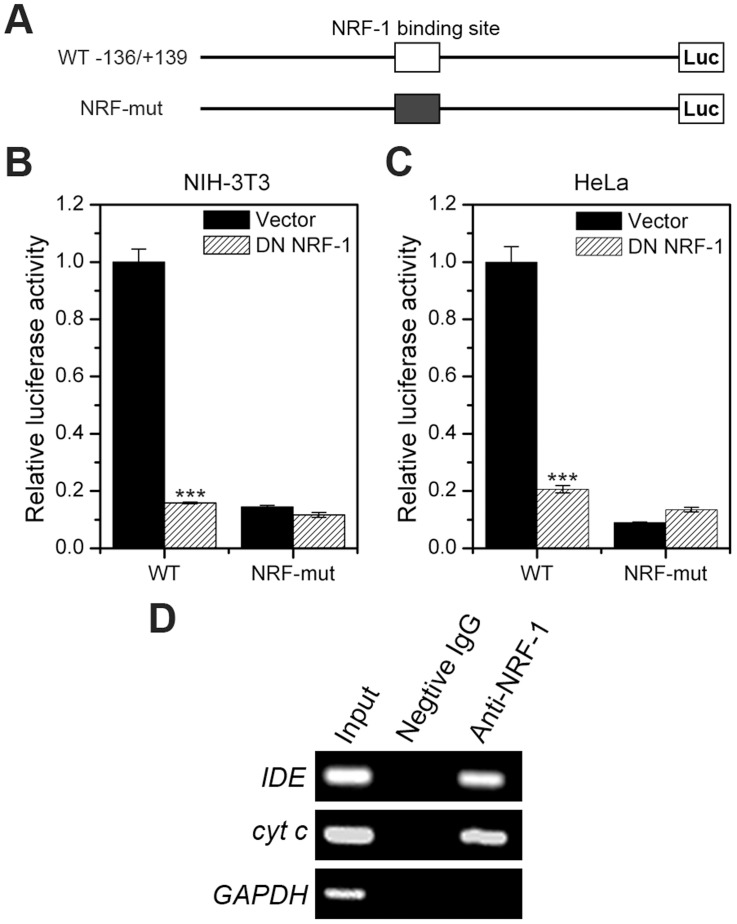
The NRF-1 binding motif in the mouse *IDE* promoter is functional. (**A**) Representation of wild-type (−136/+139 WT) and the NRF-1 binding site-mutated (NRF-mut) luciferase reporter plasmids of the mouse *IDE* promoter. (**B**) and (**C**) Dominant negative NRF-1 represses *IDE* promoter activity. NIH-3T3 and HeLa cells were transiently co-transfected with wild-type (-136/+139 WT) or NRF-1 binding site-mutated (NRF-mut) *IDE* reporter plasmids (0.4 µg) and *Renilla* luciferase plasmid (4 ng) along with or without dominant negative (DN) NRF-1 expression plasmids (0.4 µg). Twenty-four hours after transfection, cells were lysed, and the luciferase activity was examined. Firefly luminescence signal was normalized based on the *Renilla* luminescence signal. (**D**) ChIP. NRF-1 binding to the *IDE* promoter in NIH-3T3 cells was determined by ChIP. The promoter of *cytochrome c* (*cyt c*) is used as a positive control for NRF-1 binding, while the promoter of *GAPDH* acts as a negative control.

Next, we aimed to demonstrate whether this NRF-1 binding motif is functional. Mammalian cells are not sensitive to NRF-1 stimulation [25], [26]. To determine the effect of NRF-1 on *IDE* transcription, we employed a dominant negative form of NRF-1which lacks the trans-activating domain [27]. In both NIH-3T3 and HeLa cells, dominant negative NRF-1 repressed *IDE* promoter activity dramatically (Figure 4 A–C). When the NRF-1 binding motif was mutated, the effect of dominant negative NRF-1 on *IDE* transcription was abolished (Figure 4 A–C). We subsequently tested whether NRF-1 binds to the predicted NRF-1 binding motif *in vivo* by chromatin immunoprecipitation assays (ChIP). Indeed, NRF-1 binds to this site and the promoter of a positive control gene, *cytochrome c*, but not to the promoter of a negative control gene, *GAPDH* (Figure 4D). PCR products were cloned and sequenced to verify the specificity of the primers. Therefore, the NRF-1 binding motif in the mouse *IDE* core promoter is functional.

### The NRF-1 Binding Site in the *IDE* Promoter is Conserved between Mouse and Human

**Figure 5:**
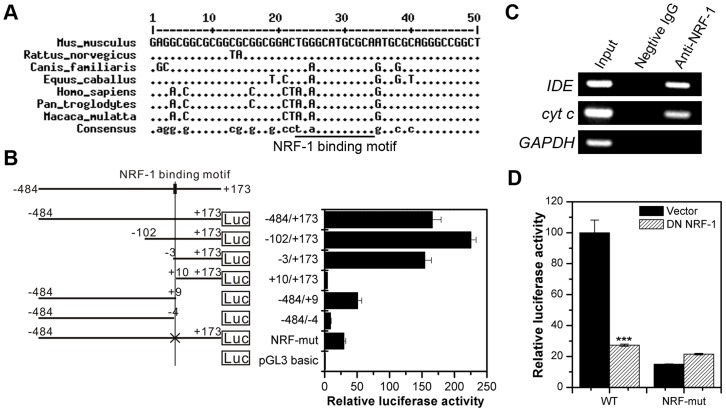
The NRF-1 binding site in the *IDE* promoter is conserved among different species. (**A**) The NRF-1 binding site in the *IDE* promoter is conserved among different species. The underlined region indicates the conserved NRF-1 binding motif. (**B**) The NRF-1 binding motif is critical for human *IDE* transcription initiation. Different truncations of the human *IDE* promoter were cloned into the pGL3-basic vector. Luciferase activity of the reporter plasmids in HeLa cells is represented as fold of the pGL3-basic vector. (**C**) ChIP. NRF-1 binding to the *IDE* promoter in HeLa cells was determined by ChIP. The promoter of *cytochrome c* (*cyt c*) is used as a positive control for NRF-1 binding, while the promoter of *GAPDH* acts as a negative control. (**D**) The NRF-1 binding motif is essential for the effect of dominant negative NRF-1 on human *IDE* promoter activity. HeLa cells were transiently co-transfected with wild-type (−484/+173) or NRF-1 binding site-mutated (NRF-mut) human *IDE* reporter plasmids (0.4 µg) and *Renilla* luciferase plasmid (4 ng) along with or without dominant negative (DN) NRF-1 expression plasmids (0.4 µg). Twenty-four hours after transfection, cells were lysed, and the luciferase activity was examined. Firefly luminescence signal was normalized based on the *Renilla* luminescence signal.

**Figure 6:**
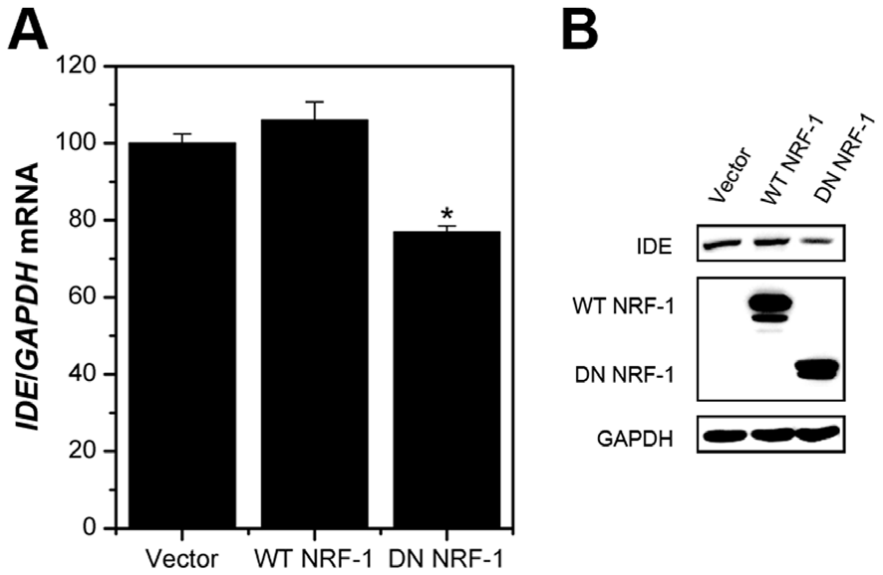
Dominant negative NRF-1 represses endogenouse IDE expression. HeLa cells were transiently transfected with wild-type (WT) or dominant negative (DN) NRF-1 expression plasmids or the empty vector (4 µg). Twenty-four hours later, total RNA were prepared and analyzed by quantitative real-time RT-PCR (**A**). *GAPDH* was used as an internal control. Alternatively, the IDE protein levels were detected by Western immublotting (**B**). GAPDH was used as an internal control. Overexpression of NRF-1 was confirmed by His-tag antibodies. *p<0.05 as compared with the vector control.

The NRF-1 binding site in the *IDE* promoter is conserved among different species (Figure 5A). The human *IDE* promoter contains a NRF-1 binding motif, which locates between −3 and +9 relative to the first transcription initiation site (Figure 5B). Deleting the 5′ terminus of the human *IDE* promoter from −484 to −3 retained its transcriptional activity, while further deletion to +10 abolished its transcriptional activity (Figure 5B). On the other hand, deleting the 3′ terminus of the human *IDE* promoter from +173 to +9 retained its transcriptional activity, while further deletion to −4 abolished its transcriptional activity (Figure 5B). Furthermore, mutation of the NRF-1 binding motif reduced the human *IDE* promoter activity by approximately 70% (Figure 6B). These results indicate that the NRF-1 binding motif is critical for human *IDE* transcription initiation. ChIP experiments showed that NRF-1 binds to the human *IDE* promoter in HeLa cells (Figure 5C). Furthermore, dominant negative NRF-1 repressed the human *IDE* promoter activity dramatically, which was abolished by the mutation of the NRF-1 binding motif (Figure 5D). Therefore, the NRF-1 binding motif in the human *IDE* promoter is functional.

To test whether NRF-1 regulates endogenous IDE expression, HeLa cells were transiently transfected with wild-type or dominant negative NRF-1 expression plasmids. Both the mRNA and protein levels of IDE were not sensitive to wild-type NRF-1 stimulation (Figure 6 A and B). However, enforced expression of dominant negative NRF-1 decreased IDE mRNA levels by 25%, and also reduced IDE protein levels, as compared with the vector control (Figure 6 A and B).

### TBP is not Essential for *IDE* Transcription Initiation

**Figure 7:**
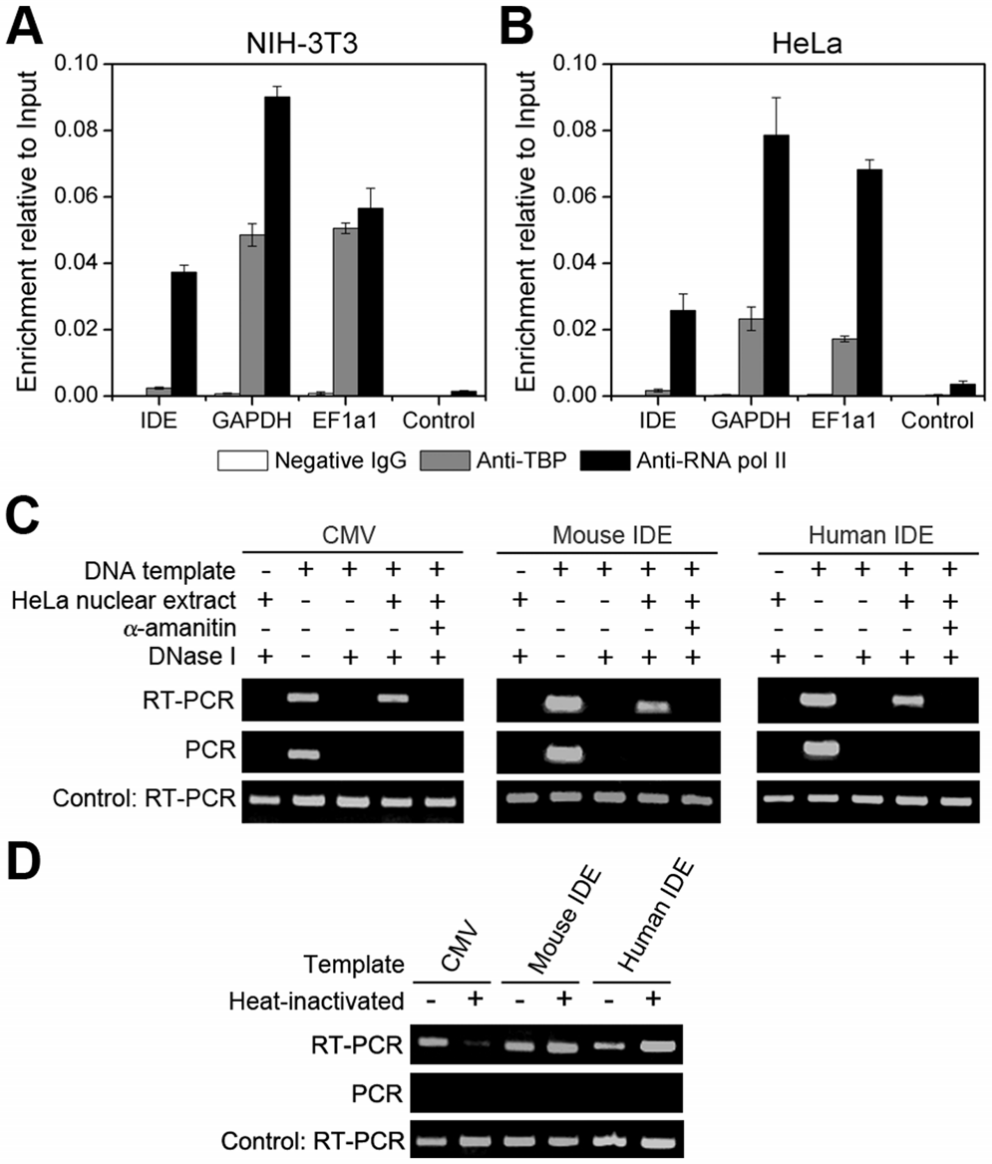
TBP is not required for *IDE* transcription initiation. (**A**) and (**B**) TBP is not associated with the *IDE* promoter. Binding of TBP and RNA polymerase II to the *IDE* promoter in NIH-3T3 (**A**) and HeLa (**B**) cells was tested by ChIP assays. Promoters of *GAPDH* and *EF1α1* were used as positive controls for TBP and RNA polymerase II binding. Negative controls were also included. Data are represented as relative enrichment to the input. (**C**) *In vitro* transcription assays. DNA template for the *CMV*, mouse *IDE* or human *IDE* promoter was incubated with HeLa nuclear extracts and ribonucleotides. The resulting transcripts were purified, digested with DNase I to eliminate the DNA template and detected by RT-PCR. An internal control RNA was included to indicate the purification efficiency of different samples. Complete elimination of the DNA template was confirmed by PCR. Transcription from all the three DNA templates proceeded only when both the DNA template and HeLa nuclear extracts existed, and was inhibited by 1 µg/mL of α-amanitin. (**D**) Heat-inactivation of TBP does not block *IDE* transcription. HeLa nuclear extracts were heated at 47°C for 15 min before *in vitro* transcription assays.

The *IDE* promoter is CpG-rich and contains no TATA box. Therefore, we wondered whether TBP is required for *IDE* transcription initiation. ChIP assays showed that TBP is not associated with the *IDE* promoter in both NIH-3T3 (Figure 7A) and HeLa (Figure 7B) cells. By contrast, TBP is recruited to the promoters of *GAPDH* and *elongation factor 1 α 1* (*EF1α1*), which contain the TATA box. These results suggest that TBP is not essential for *IDE* transcription initiation.

To further address this question, we carried out *in vitro* transcription assays. The *CMV* promoter, which contains the TATA box, was used as a control. *In vitro* transcription occurred from all three DNA templates of the *CMV*, mouse *IDE* and human *IDE* promoters, which was abolished by α-amanitin (Figure 7C). When the DNA template or the nuclear extract was omitted, no transcripts were detected (Figure 7C). TBP was reported to be specifically inhibited by being heated at 47°C for 15 minutes, which was rescued by adding back of TBP [28]. *In vitro* transcription assays showed that transcription from the *CMV* promoter was inhibited by heat-inactivation of TBP, while transcription from the mouse or human *IDE* promoters was activated (Figure 7D), indicating that TBP is not required for *IDE* transcription initiation.

## Discussion

Despite the prevalence of CpG islands associated with mammalian promoters, the mechanism of transcription initiation from this type of promoters is not well understood. CpG island promoters often lack the TATA box or other common core promoter elements. It has been proposed that Sp1 may participate in the transcription initiation of CpG island promoters, because Sp1 binds to a consensus sequence of GGGCGG which is present in many CpG islands due to their high GC content [29]. NRF-1 is a transcription factor that activates nuclear genes required for respiration as well as genes regulating heme biosynthesis and mitochondrial DNA transcription and replication. NRF-1 has been reported to play an important role in the transcriptional regulation of several genes [30], [31], [32], [33]. Interestingly, NRF-1 binding motifs are frequently found around the transcription initiation sites of genes [27], [34], typically those without the TATA box [35], suggesting that NRF-1 may function in regulating the transcription initiation of TATA box-less genes.

Here we studied the mechanism of transcription initiation of the ubiquitously expressed gene, *IDE*, which has a CpG island promoter and dispersed transcription initiation sites. We mapped the core promoter region of *IDE*, and showed that the *IDE* core promoter contains a NRF-1 binding motif that is essential for transcription initiation. We further demonstrated that NRF-1 binds to this motif and dominant negative NRF-1 represses *IDE* transcription. Our studies suggest that, besides Sp1, NRF-1 may be another important transcription factor that regulates the transcription initiation from CpG island promoters. These results are suggestive for the mechanism of transcription initiation from other CpG island promoters. Since CpG island promoters often lack canonical core promoter elements, some transcription factors, such as Sp1 and NRF-1, may function in recruiting the transcription apparatus to the promoter region without the assistance of other core promoter elements. In this hypothesis, the transcription apparatus is not “fixed” to the promoter through its own interactions to the promoter, which may explain the fact that CpG island promoters often have dispersed transcription initiation sites.

TBP was originally regarded as a universal factor that participated in the transcription initiation of genes transcribed by all three RNA polymerases in eukaryotes [36]. However, emerging studies have indicated the existence of TBP-independent transcription by RNA polymerase II [37], [38]. In addition, A TBP-free TAF_II_-containing complex was reported to initiate transcription from both TATA box-free and TATA box-containing promoters [39]. The *IDE* promoter contains no TATA box, and is free of TBP binding. Furthermore, inactivation of TBP does not block *IDE* transcription. These results suggest that TBP is not required for *IDE* transcription initiation. It is fascinating to demonstrate whether TBP is essential for the transcription initiation from other CpG island promoters which do not contain the TATA box.

IDE is a zinc metalloprotease that typically degrades insulin and Aβ, and is associated with both type II DM and AD. However, the mechanism of the transcriptional regulation of *IDE* has not been studied in detail. Here, we showed that NRF-1 acts as a central transcription factor that regulates *IDE* transcription. Thus, NRF-1 dysfunction may result in a down-regulation of IDE expression and DM or AD pathogenesis. Indeed, human genetic studies suggest that NRF-1 polymorphisms are associated with the pathogenesis of type II DM [40], [41]. The transcriptional regulation of *IDE* by NRF-1 reported here provides a mechanism that links NRF-1 to type II DM. Although IDE is predominantly distributed in the cytosol, an isoform of IDE generated by alternative translation initiation is targeted to mitochondria [42]. NRF-1 is an important transcription factor that mediates nuclear-mitochondrial interactions. The transcriptional regulation of *IDE* by NRF-1 may be of significance to the mitochondrial function.

In conclusion, our studies indicate that NRF-1 mediates *IDE* transcription initiation in a TBP-independent manner, and provide insights into the potential mechanism of the transcription initiation from other CpG island promoters. NRF-1 has been reported to regulate the transcription of a variety of genes. It is fascinating to investigate whether NRF-1 is essential for the transcription initiation of these genes. In addition, NRF-1 interacts with the peroxisome proliferator-activated receptor γ coactivator 1 (PGC-1) family of proteins, including PGC-1α [43], PGC-1β [44] and PGC-1-related coactivator (PRC) [45]. It remains to be clarified whether NRF-1 regulates *IDE* transcription initiation through these coactivators.

## Materials and Methods

### Plasmid Constructs

Genome DNA from C57/BL6 mice or HeLa cells was used as the template to construct the promoter reporter plasmids. Different truncations of the mouse and human *IDE* promoter were cloned into the *Xho* I and *Hind* III restriction sites of the pGL3-basic vector (Promega, Madison, WI). The primers used during the plasmid construction are shown in Table S1 and S2. The NRF-1 binding sites in the mouse and human *IDE* promoter were mutated from TGGGCATGCGCA and AGAGCATGCGCA to TGGGCAGCGACA and AGATAGGTAGCA, respectively. The expression plasmids for wild-type NRF-1and dominant negative NRF-1 which contains 1–304 amino acids of full-length NRF-1 [27] were constructed by cloning the corresponding sequences into the *Bam* HI and *Xba* I restriction sites of the pcDNA 3.1 myc-His A vector (Invitrogen, Carlsbad, CA).

### Cell Culture and Cell Transfection

NIH-3T3 and HeLa cells were maintained in Dulbecco’s modified Eagle’s medium (Thermo Scientific HyClone, Logan, UT) containing 10% fetal bovine serum (Thermo Scientific HyClone). HeLa cells were seeded into 6-well plates at a density of 4×10^5^ cells per well in antibiotic-free medium the day before transfection. Each well of cells were transiently transfected with 4 µg of pcDNA 3.1, wild-type or dominant negative NRF-1 expression plasmids using Lipofectamine™ 2000 (Invitrogen). Twenty-four hours later, cells were collected, and analyzed by quantitative real-time RT-PCR or Western immunoblotting.

### 5'-RACE

5′-RACE was carried out using the SMART™ RACE cDNA Amplification Kit (Clontech, Shiga, Japan) according to the manufacturer’s instructions, to determine the transcription initiation sites of mouse *IDE*. 5′-RACE-ready cDNA was synthesized from total RNA from NIH-3T3 cells. The 5′-flanking region of *IDE* was amplified using the universal primer A mix (Clontech) and an *IDE*-specific primer: 5′-TCTTCAACGTGCAATAACCCT-3′. The resulting PCR products were used as a template for a nested PCR reaction using the nested universal primer A (Clontech) and a nested *IDE*-specific primer: 5′-TGTGGTGGGATCGCTGATGAGAAG-3′. The nested PCR products were then cloned into the pMD19-T vector (Takara Bio Inc., Shiga, Japan), and a total of 33 positive clones were sequenced.

### Dual-luciferase Reporter Assays

NIH-3T3 and HeLa cells were seeded into 24-well plates at a density of 8×10^4^ cells per well in antibiotic-free culture medium the day before transfection. Each well of cells were transiently co-transfected with 0.8 µg of *IDE* reporter plasmids and 8 ng of *Renilla* reporter plasmid (pCMV-RL, Promega) as an internal control using Lipofectamine™ 2000. Alternatively, cells were co-transfected with 0.4 µg of *IDE* reporter plasmids, 4 ng of pCMV-RL plasmid and 0.4 µg of pcDNA 3.1 or NRF-1 expressing plasmids. Twenty-four hours later, cells were lysed and the luciferase acitvity was detected using the Dual-Luciferase Reporter Assay System (Promega). Firefly luminescence signal was normalized based on the *Renilla* luminescence signal.

### RNA Isolation and Quantitative Real-time RT-PCR

RNA was isolated using Trizol reagent (Invitrogen), and treated with DNase I (Promega) before cDNA synthesis. cDNA was synthesized with 2 µg of total RNA with anchored oligo (dT)_20_ primers using the Transcript First-Strand cDNA Synthesis Kit (Transgene Biotechnology Inc., Beijing, China). Quantitative real-time PCR was performed using the UltraSYBR mixture (Cwbiotech, Beijing, China) in the Stratagene Mx3000P™ Real-Time PCR System (Agilent Technologies, La Jolla, CA). PCR primers for human *IDE* were forward: 5′-AAAGACAAAGAGAGGCCACGGGG-3′ and reverse: 5′-TGGCAACCCGGACATTTTCTGGTC-3′. PCR primers for human *GAPDH* were forward: 5′-ACCGTCAAGGCTGAGAACGGGA-3′ and reverse: 5′-CCTGCAAATGAGCCCCAGCCTT-3′. Both the *IDE* and *GAPDH* primers are intron-spanning. Melting curves were performed on the products, which confirmed the specificity of the primers. The thermal cycling conditions were as follows: initial denaturation at 95°C for 10 min, followed by 40 cycles of denaturing at 95°C for 15 s, annealing at 60°C for 30 s and extension at 72°C for 30 s.

### Western Immunoblotting

Cells were collected and lysed with the cell lysis buffer (Beyotime, Shanghai, China). Protein concentrations were determined using the BCA method. Whole cell extracts were boiled for 4 min, resolved by 10% SDS-PAGE and electrophoretically transferred to polyvinylidene difluoride membranes (Millipore, Schwalbach, Germany). The membranes were blocked with 5% non-fat dry milk in Tris-buffered saline containing 0.05% Tween 20 at room temperature for 1 h, and then incubated with mouse monoclonal anti-IDE (1∶1000, Abcam), mouse monoclonal anti-His tag (1∶2000, Cwbiotech) or rabbit polyclonal anti-GAPDH (1∶1000, Cwbiotech) antibodies at 4°C overnight, followed by an incubation with the appropriate horseradish peroxidase-conjugated secondary antibodies (1∶10000, Cwbiotech) at room temperature for 1 h. The chemiluminescence reaction was performed using ECL reagent (Thermo Scientific).

### ChIP

ChIP was performed using the Magnetic Chromatin Immunoprecipitation Kit (Active Motif, Carlsbad, CA) as described in the manufacturer’s protocol. Briefly, NIH-3T3 or HeLa cells were washed once with phosphate buffered saline, and fixed in culture medium containing 1% formaldehyde at room temperature for 10 min. The cells were then collected and lysed to release the nucleus. The nucleus was digested with the enzyme mix at 37°C for 10 min to shear the chromatin into small segments. The sheared chromatin was then immunoprecipitated with 2 µg of NRF-1 antibodies (Abcam), 2 µg of TBP antibodies (Abcam), 2 µg of RNA polymerase II antibodies (Active Motif), or a negative control IgG at 4°C for 4 h. The pulled-down chromatin was washed, reverse-crosslinked and purified. Semi-quantitative or quantitative real-time PCR was performed using the primers shown in Table S3.

### *In vitro* Transcription

*In vitro* transcription was performed using the HeLaScribe® Nuclear Extract in vitro Transcription System (Promega) with some modifications. DNA template of the mouse *IDE* promoter was amplified from mouse genomes using primers forward: 5′-TGCCGAGACGACGACCCACC-3′ and reverse: 5′-TGAGAAGCGGTTTCCCACGAC-3′. DNA template of the human *IDE* promoter was amplified from human genomes using primers forward: 5′- CTCACAGTCAGACACACGTCGCCACC -3′ and reverse: 5′-CCACACGGTCCTGGAAACTCAGTGCC-3′. DNA template of the *CMV* promoter was amplified from a mouse peroxisome proliferator-activated receptor α expression plasmid drived by the *CMV* promoter using primers forward: 5′-TGTTGGAGGTCGCTGAGTAGTGC-3′ and reverse: 5′-AGGCGGGTTGTTGCTGGTCT-3′. The DNA template (100 ng) was incubated with HeLa nuclear extracts and four types of ribonucleotides at 37°C for 30 minutes. The resulting transcripts were purified, digested with DNase I (Promega) at 37°C for 1 h to eliminate the DNA template, and then used as RT-PCR template. An internal control RNA was included to indicate the purification efficiency of different samples. RT-PCR was performed using the PrimeScript® One Step RT-PCR Kit Ver.2 (Takara), using the following primers–forward: 5′-GCACCTTGCGCTCCATCCTCG-3′ and reverse: 5′-CCGCTGCACTTGTGGCGTTCTC-3′ for mouse *IDE*, forward: 5′-CCAGCACCTTCCGCTCAGTCC-3′ and reverse: 5′-GGTGACTCTGTCCGCCCCTGC-3′ for human *IDE*, and forward: 5′-GACCTGGAAAGTCCCTTATCT-3′ and reverse: 5′-AGCCCTTACAGCCTTCACAT-3′ for *CMV*. Elimination of DNA template was confirmed by PCR. To confirm that *IDE* is transcribed by RNA polymerase II, 1 µg/mL of α-amanitin was included in the *in vitro* transcription system. To determine the role of TBP in transcription initiation, HeLa nuclear extracts were heated at 47°C for 15 min before the *in vitro* transcription assay.

### Statistical Analysis

Results are represented as means ± S.E.M from three independent experiments. Statistical significance was determined by one-way ANOVA, followed by the post-hoc Tukey multiple comparison test.

## Supporting Information

Table S1 **PCR primers used for constructing the reporter plasmids of the mouse** ***IDE*** **promoter.**
(DOC)

Table S2 **PCR primers used for constructing the reporter plasmids of the human** ***IDE*** **promoter.**
(DOC)

Table S3 **PCR primers used for ChIP assays.**
(DOC)
