# Supplementary material for: Nuclear Respiratory Factor 1 Mediates the Transcription Initiation of Insulin-Degrading Enzyme in a TATA Box-Binding Protein-Independent Manner
Source: PLoS One. 2012 Aug 3;7(8):e42035. doi: 10.1371/journal.pone.0042035 (PMC3411688; doi:10.1371/journal.pone.0042035)
Supplement: Table S1 — PCR primers used for constructing the reporter plasmids of the mouse IDE promoter. (DOC) [file pone.0042035.s001.doc]

Table S1 PCR primers used for constructing the reporter plasmids of the mouse IDE promoter

| **Primer^a^** | **Sequence (5' to 3')^b^** |
| --- | --- |
| Forward primers |  |
| F-2274 | TAATGCCAGCCTGCCACCACTG |
| F-1636 | GCAGTCGGGTGCTCCTACCCAC |
| F-1236 | TCCAGCCTAGGGCTCTGAGCAT |
| F-635 | ACTTTGTGCTGGTGTGGGGGAG |
| F-136 | TGCCGAGACGACGACCCACC |
| F-64 | GTAAAACCCGGAGCGGAGATCG |
| F-23 | CGGCGCGGCGCGGCGGACT |
| F +7 | AATGCGCAGGGCCGGCTTCC |
| Reverse primers |  |
| R+139 | CGAGGATGGAGCGCAAGGTG |
| R+75 | ACCCCGGCGCAGCCGCTG |
| R+48 | CGAACGCTTCCTTTTGGCGCTG |
| R+24 | AAGCCGGCCCTGCGCATT |
| R +9 | ATTGCGCATGCCCAGTCC |
| R -6 | GTCCGCCGCGCCGCGCCGCCT |

^a^The number indicates the position of the 5' terminus of the primer relative to the first transcription initiation site of mouse IDE.

^b^The primer sequences do not include the restriction sites and the protection bases.
