# Supplementary material for: Nuclear Respiratory Factor 1 Mediates the Transcription Initiation of Insulin-Degrading Enzyme in a TATA Box-Binding Protein-Independent Manner
Source: PLoS One. 2012 Aug 3;7(8):e42035. doi: 10.1371/journal.pone.0042035 (PMC3411688; doi:10.1371/journal.pone.0042035)
Supplement: Table S2 — PCR primers used for constructing the reporter plasmids of the human IDE promoter. (DOC) [file pone.0042035.s002.doc]

**Table S2 PCR primers used for constructing the reporter plasmids of the human IDE promoter**

| **Primer^a^** | **Sequence (5' to 3')** ^b^ |
| --- | --- |
| Forward primers |  |
| F-484 | CCTTGTCTCCAGCGCATCTGT |
| F-102 | TCACCGTCCTCGCCTGCGTCCT |
| F-3 | AGAGCATGCGCAGTGCGCAGG |
| F+10 | GTGCGCAGGGCCGGCTCGAAG |
| Reverse primers |  |
| R+173 | CACACAGGCGCTCCGGAGGCG |
| R+9 | TGCGCATGCTCTAGCCGCGGCG |
| R-4 | AGCCGCGGCGCCGCGCGGTCT |

^a^The number indicates the position of the 5' terminus of the primer relative to the first transcription initiation site of human IDE.

^b^The primer sequences do not include the restriction sites and the protection bases.
