# Supplementary material for: Nuclear Respiratory Factor 1 Mediates the Transcription Initiation of Insulin-Degrading Enzyme in a TATA Box-Binding Protein-Independent Manner
Source: PLoS One. 2012 Aug 3;7(8):e42035. doi: 10.1371/journal.pone.0042035 (PMC3411688; doi:10.1371/journal.pone.0042035)
Supplement: Table S3 — PCR primers used for ChIP assays. (DOC) [file pone.0042035.s003.doc]

Table S3 PCR primers used for ChIP assays

| **Primer** | **Sequence (5' to 3')** | **Product length (bp)** |
| --- | --- | --- |
| Mouse IDE |  | 139 |
| Forward | GTAAAACCCGGAGCGGAGATCG |
| Reverse | ACCCCGGCGCAGCCGCTG |
| Mouse cytochrome c |  | 205 |
| Forward | CTGTCGTAAGTGTCGGGCAAAC |
| Reverse | GCCTATGTACCGGTGTCGGTG |
| Mouse GAPDH |  | 161 |
| Forward | GCCCTTGAGCTAGGACTGGAT |
| Reverse | CCTGGCACTGCACAAGAAGAT |
| Mouse EF1α1 |  | 159 |
| Forward | ATAAGTGCGGCAGTCGCCTTGG |
| Reverse | AATGCTCGCAGCTAATCCCCGC |
| Mouse Control |  | 218 |
| Forward | AGAGTACCGACTGTTCTTCCG |
| Reverse | GCTTTATTGTGCCCCTAGTGT |
| Human IDE |  | 162 |
| Forward | GGCCAAACCCCTGTCCCTCCA |
| Reverse | CGCATTAGCCAGCGCAGTCGC |
| Human cytochrome c |  | 174 |
| Forward | CTCGAGAAGAAGGGCACGCACG |
| Reverse | AGTGCAACCAAACTCGCTCCGC |
| Human GAPDH |  | 224 |
| Forward | AAAGCGGGGAGAAAGTAGGGC |
| Reverse | CCTGGCGACGCAAAAGAAGAT |
| Human EF1α1 |  | 206 |
| Forward | GGTAAACTGGGAAAGTGATGTCG |
| Reverse | GCGTGGAAGTAATTCAAGGCA |
| Human Control |  | 155 |
| Forward | TCCCACCTAGCAGCAAGCACAG |
| Reverse | TGCCCAGCCTGCTGACTGTATT |
